# Electrochemical corrosion behavior of cast and SLM Co-Cr dental alloys in artificial saliva with variable pH

**DOI:** 10.25122/jml-2025-0130

**Published:** 2025-11

**Authors:** Alexandra Elena Biculescu, Anca Iuliana Popescu, Ioana Ana Maria Ciorniciuc, Ruxandra Nicolette Voinea-Georgescu, Raluca Monica Comăneanu, Costin Coman, Anca Monica Dobrescu

**Affiliations:** 1 Doctoral School of Dental Medicine, Titu Maiorescu University, Bucharest, Romania; 2 Faculty of Dental Medicine, Titu Maiorescu University, Bucharest, Romania

**Keywords:** Cobalt-Chromium alloys, Selective Laser Melting, casting, corrosion resistance, artificial saliva

## Abstract

This study compares the corrosion resistance of two cobalt–chromium (Co–Cr) dental alloys manufactured by casting (Wirobond SG) and Selective Laser Melting (Mediloy S-Co). Standardized disc specimens were analyzed morphologically (SEM), elementally (EDS), and electrochemically (OCP and Tafel plots). Tests were performed in Carter-Brugirard artificial saliva (pH 4.9 and 6.8) at 37 ± 1°C. Electrochemical parameters (E_oc_, E_cor_, i_cor_, β_a_, β_c_, R_p_) were used to evaluate corrosion behavior. The cast alloy showed the highest R_p_ (1346.29 kΩ•cm^2^) and lowest icor (30.96 nA/cm^2^) at pH 6.8, indicating better corrosion resistance. SEM showed process-related surface characteristics, including porosities in SLM samples and carbide precipitates in cast samples. These are components of the material itself that make it harder and more resistant to wear. Corrosion resistance is influenced by both manufacturing method and pH. Cast Co-Cr performed better in near-neutral saliva, while the SLM alloy showed a more electropositive open-circuit potential (E_oc = 67.9 mV) at pH 4.9, indicating higher corrosion resistance from an electrochemical point of view under acidic challenge. Both materials are suitable for dental applications.

## INTRODUCTION

Cobalt-Chromium (Co-Cr) alloys have long been utilized in fixed prosthodontics due to their excellent mechanical characteristics [1], biocompatibility [2,3], and corrosion resistance in the oral cavity. New opportunities in the design and manufacturing of dental frameworks have emerged with the development of additive manufacturing technologies such as selective laser melting (SLM). In contrast to traditional casting methods, SLM offers greater design and material efficiency; however, variations in microstructure and surface integrity may affect long-term clinical performance, particularly corrosion resistance [4,5].

Corrosion resistance is essential for the clinical efficacy of dental alloys, as deterioration in the oral environment can lead to ion release, loss of structural integrity, and biological problems [6]. Artificial saliva with regulated pH is often used to replicate oral conditions in laboratory research. Assessing the electrochemical behavior of dental metals across diverse pH values provides insights into their stability under different clinical conditions.

Previous research has highlighted that the fabrication technique—casting versus SLM—can influence not only the microstructure but also the tribo-corrosion behavior of Co-Cr dental alloys [7,8]. Nonetheless, few studies have directly evaluated their electrochemical behavior under controlled acidic and near-neutral environments.

The present study seeks to evaluate the corrosion resistance of two Co-Cr alloys—one produced via casting and the other via SLM—using Scanning Electron Microscopy (SEM), Energy Dispersive X-ray Spectroscopy (EDS), and electrochemical testing, including potentiodynamic polarization in artificial saliva at pH 4.9 and 6.8. Two pH values were chosen: pH 6.8 to reflect nearly neutral resting saliva and pH 4.9 to mimic clinically significant acidic challenges (e.g., cariogenic biofilm activity or reflux episodes) that may alter corrosion susceptibility.

The present study offers a side-by-side comparison of cast vs SLM Co-Cr under two clinically significant pH settings in Carter–Brugirard saliva, correlating microstructural characteristics with E_oc_/E_corr_, i_corr_, and R_p_, and emphasizing a pH-dependent crossover with clinical ramifications.

## Material and Methods

For this study, two commercially sold Co-Cr dental alloys—both produced by Bego (Germany)—were used. Conventional casting produced the first alloy, Wirobond SG (CCT), whereas selective laser melting (SLM), an additive manufacturing method, produced the second alloy, Mediloy S-Co (CCP). The two alloys were chosen from the same manufacturer (BEGO, Germany) to distinguish between the production processes (casting vs. SLM) and to reduce differences in composition and supplier. Table 1 lists the chemical compositions supplied by the company.

**Table 1:** Chemical composition (wt%) of the tested Co-Cr alloys (manufacturer-reported nominal values)

Sample	Co	Cr	Mo	W	Si	C
CCT	63.8	24.8	5.1	5.3	1.0	-
CCP	63.9	24.7	5.0	5.4	1.0	-

The disc specimens (15 mm in diameter and 5 mm thick) were put in epoxy, leaving an area of around 1.0 cm^2^ open. Using SiC papers (P320→P1000), the surfaces were ground down and then polished using a 1 μm Al_2_O_3_ solution. The samples were then ultrasonically cleaned in ethanol and deionized water for 5 minutes each, air-dried, and their edges were sealed to prevent crevice corrosion artifacts. No post-build heat treatment was applied unless otherwise specified.

**Figure 1:**
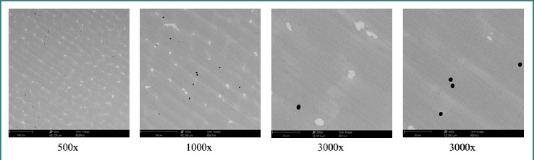
SEM morphology of the cast Co–Cr alloy at different magnifications

**Figure 2:**
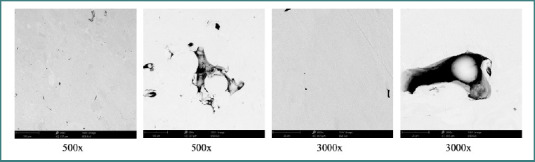
SEM morphology of the SLM Co–Cr alloy at different magnifications

Scanning electron microscopy was performed for morphological assessment using a Phenom ProX desktop SEM (Phenom World, Netherlands). The surface morphology was analyzed at magnifications ranging from 500× to 3000× (Figures 1 and 2).

**Figure 3:**
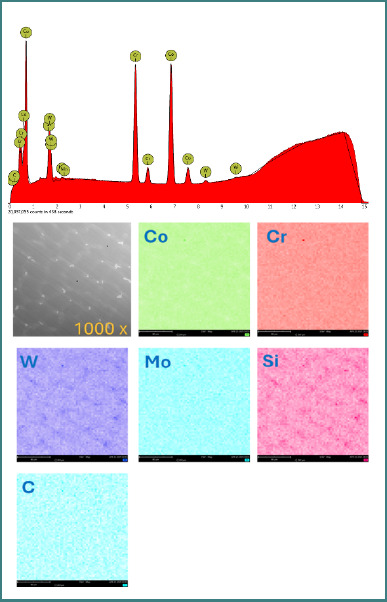
EDS analysis of the cast Co–Cr alloy (CCT) showing elemental composition and uniform distribution of Co, Cr, W, Mo, Si, and C

**Figure 4:**
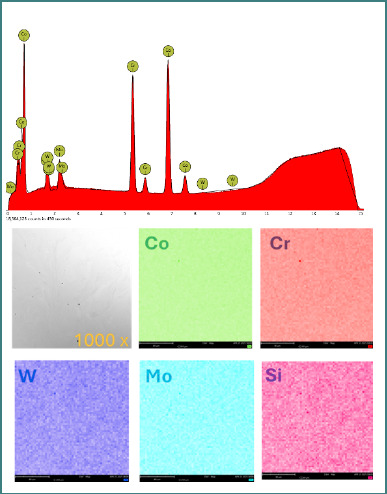
EDS analysis of the SLM alloy (CCP) showing elemental composition and uniform distribution of Co, C, W, Mo and Si

EDS integrated into the SEM device was used to examine elemental composition. For both samples, spectra and element distribution maps were tracked (Figures 3 and 4).

A PARSTAT 4000 potentiostat/galvanostat (Princeton Applied Research, USA) was used in electrochemical corrosion testing. Tests were conducted at 37 ± 1 °C in Carter–Brugirard artificial saliva, adjusted to pH 4.9 and 6.8 by drop-wise addition of 1 M HCl. Table 2 lists the electrolyte composition (g•L^-1^).

**Table 2:** Composition of Carter-Brugirard artificial saliva

Component	Amount(g/L)
Na_2_HPO_4_	0.19
NaCl	0.7
KSCN	0.33
KH_2_PO_4_	0.26
NaHCO_3_	1.5
Urea	1.3

The open-circuit potential (OCP) was continuously monitored over a 6-hour immersion period. Subsequently, potentiodynamic polarization curves (Tafel plots) were recorded at a scan rate of 0.167 mV/s by sweeping the potential from −0.2 V to +0.2 V relative to the OCP. Corrosion measurements were performed using a standard three-electrode electrochemical cell, consisting of a Teflon holder containing the test specimen as the working electrode, a platinum plate as the counter electrode, and a saturated calomel electrode (SCE) as the reference electrode.

The following electrochemical parameters were extracted from the Tafel plots: anodic slope (β_a_), cathodic slope (β_c_), polarization resistance (R_p_), corrosion potential (E_cor_), and corrosion current density (i_cor_). Following ASTM G59-97 (2014), R_p_ was computed with the Stern–Geary equation.

## Results

### Surface morphology (SEM analysis)

The surface morphology of the two tested alloys, as illustrated in Figures 1 and 2 (see Materials and Methods), reflects the influence of the respective manufacturing processes.

The cast alloy (CCT) displayed relatively uniform surfaces with minor imperfections. At higher magnification, bright formations were observed, associated with tungsten carbides (WC), which contribute to increased hardness and wear resistance. Some microporosity was also observed, likely due to the casting process.

In contrast, the SLM alloy (CCP) exhibited characteristic defects, including large pores (up to ~200 µm) and partially unmelted spherical Co-Cr particles, typical of the SLM process. These defects may affect passive layer formation and electrochemical stability.

### Elemental composition (EDS analysis)

Elemental analysis by EDS confirmed the presence of the main alloying elements in both materials. Small variations in Mo and W concentrations were observed. Carbon was detected only in the cast alloy (CCT) (Table 3).

**Table 3:** Chemical composition (wt%) of the studied Co-Cr alloys (EDS-measured values)

Sample	Co	Cr	Mo	W	Si	C
CCT	61.8	23.82	6.12	6.81	149	0.08
CCP	62.87	24.06	4.87	6.29	1.91	-

### Open circuit potential (OCP)

**Figure 5:**
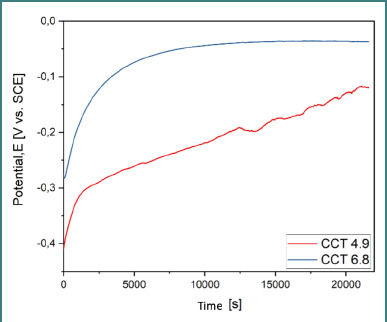
OCP evolution for CCT at pH 4.9 and 6.8

**Figure 6:**
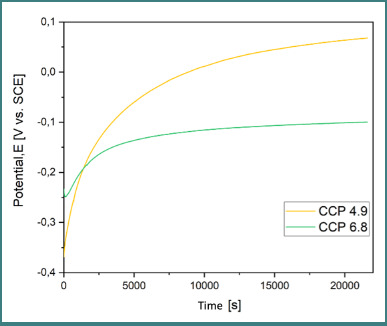
OCP evolution for CCP at pH 4.9 and 6.8

**Figure 7:**
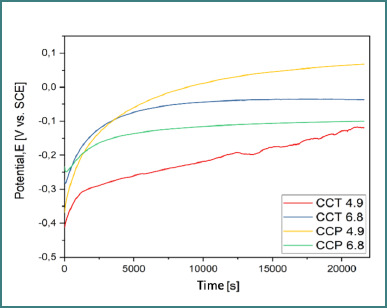
Comparative OCP evolution for both samples in both pH conditions

OCP was recorded over 6 hours in artificial saliva at two pH values (4.9 and 6.8). The cast alloy (CCT) (Figure 5) showed more stable potential shifts, indicating greater stability from an electrochemical perspective. The SLM alloy (CCP) (Figures 6 and 7) demonstrated the most electropositive potential (67.9 mV) in acidic conditions (pH 4.9) (Table 4).

**Table 4:** Surface morphology (SEM summary) and stabilized open-circuit potential (E_oc_, mV vs. SCE)

Alloy	Process	SEM key features	E_oc_ at pH 6.8(mV)	E_oc_ at pH 4.9(mV)
Wirobond SG (CCT)	Casting	Dendritic matrix; interdendritic carbides	−37.1	−118.8
Mediloy S-Co (CCP)	SLM	Melt-pool bands; fine cellular/dendritic substructure; pores/particulates	−99.8	+67.9

### Potentiodynamic polarization (Tafel analysis)

**Figure 8:**
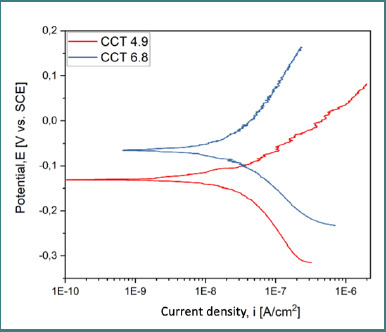
Tafel curves for CCT at pH 4.9 and 6.8

**Figure 9:**
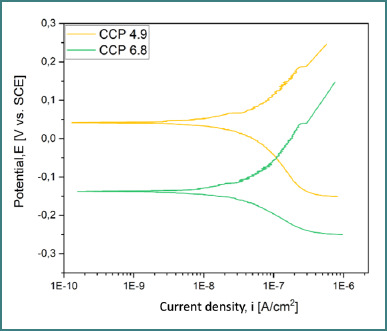
Tafel curves for CCP at pH 4.9 and 6.8

**Figure 10:**
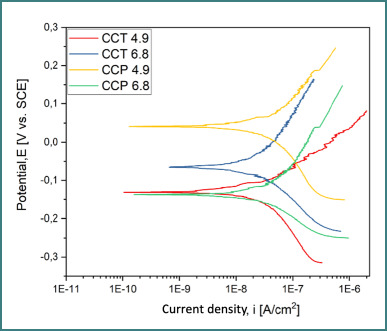
Overlay of all Tafel curves for both samples

Tafel curves (Figures 8-10) provided data on corrosion potential (E_cor_), corrosion current density (i_cor_), anodic and cathodic slopes (β_a_, β_c_), and polarization resistance (R_p_). The best corrosion resistance was observed for the cast alloy (CCT) (Figure 8) at pH 6.8, while the SLM alloy (CCP) (Figure 9) had the most electropositive E_cor_ at pH 4.9 (Table 5).

**Table 5:** Electrochemical parameters extracted from Tafel plots

Sample	pH	E_oc_(mV)	E_cor_(mV)	i_cor_(nA/cm^2^)	β_c_ (mV•dec^-1^)	β_a_ (mV•dec^-1^)	R_p_ (kΩ•cm^2^)
CCT	4.9	-118.8	-127.2	33.55	223.85	114.51	981.61
CCT	6.8	-37.1	-63.9	30.96	151.72	260.49	1346.29
CCP	4.9	67.9	42.2	51.81	241.17	195.47	905.93
CCP	6.8	-99.75	-138.9	46.06	124.15	231.42	762.65

## Discussion

The corrosion resistance of cobalt-chromium alloys used in fixed prosthodontics is influenced by several related factors, including manufacturing technique, chemical composition, microstructural homogeneity, and the electrochemical stability of the passive oxide layer. The present study compared the behavior of two Co-Cr alloys—one cast and the other fabricated via SLM—when immersed in artificial saliva at two pH values to evaluate their suitability for use in variable oral environments.

The superior corrosion resistance of the cast alloy (CCT) in near-neutral saliva (pH 6.8), demonstrated by the highest polarization resistance (R_p_ = 1346.29 kΩ•cm^2^) and the lowest corrosion current density (i_cor_ = 30.96 nA/cm^2^), aligns with previous studies emphasizing the role of passive film stability in alkaline environments [9,10]. Cast Co-Cr alloys generally benefit from a denser surface and more uniform carbide distribution, particularly of tungsten and molybdenum carbides, which may contribute to passivation by limiting active surface exposure [11,12].

In contrast, the SLM alloy (CCP) showed a more electropositive corrosion potential at pH 4.9 (E_cor_ = 42.2 mV), suggesting enhanced corrosion resistance from an electrochemical perspective in acidic environments. This effect may be attributed to local microstructural features and internal stress patterns induced by the additive process, which, in some studies, were shown to positively influence corrosion behavior under specific conditions [13]. However, the presence of surface porosities and unmelted Co-Cr particles, as revealed by SEM analysis, suggests that the laser-melting process can introduce morphological imperfections that compromise oxide-layer formation and adhesion [11,14,15].

It is worth noting that while SLM techniques enable precise geometric control and digital integration in CAD/CAM workflows, their electrochemical performance is highly dependent on post-processing steps. Heat treatment, surface finishing, and optimization of laser parameters can all reduce residual porosity and improve corrosion behavior, as shown in other recent investigations [8,13]. Without these interventions, surface defects can act as initiation sites for localized corrosion, particularly in near-neutral or slightly alkaline pH, where passivation is expected to dominate.

EDS analysis confirmed small but relevant differences in chemical composition between the two alloys. Notably, the cast alloy showed a slightly higher Mo and W content, while CCP had no detectable carbon. The presence of Mo is known to enhance corrosion resistance by promoting stable oxide formation, particularly in acidic environments, which may partially explain CCP’s E_cor_ advantage at low pH [9,10]. Moreover, the absence of iron (Fe) in both alloys is consistent with improved corrosion resistance, as Fe is often associated with lower electrochemical stability in oral conditions [12].

The correlation between increasing pH and decreasing i_corr_, observed in both alloys, supports the established understanding that an alkaline environment favors passivation, while acidic conditions tend to dissolve oxide films or prevent their formation [9,10,16]. Similar results have been reported in in vitro studies simulating saliva, where Co-Cr alloys showed increased corrosion resistance with rising pH [16,17].

From a clinical perspective, the findings of this study suggest that both fabrication methods are viable, but material selection should consider the patient’s oral pH, prosthesis location, and the potential for salivary pH fluctuations. Cast Co-Cr alloys remain a reliable option for oral cavities with relatively stable pH near neutrality, while SLM alloys could be appropriate for patients with mild to moderate acidic conditions, provided that proper surface optimization is ensured.

This study presents short-term electrochemical evaluations in static artificial saliva (two pH levels), devoid of mechanical loads, fluoride, or tribocorrosion. The evaluation was limited to two alloys from a single producer. Surface finishing was standardized for comparison, but it may not reflect all clinical processes. These limitations restrict direct in vivo extrapolation.

## CONCLUSION

Within the limitations of this in vitro study, the following conclusions can be drawn:

- The cast Co-Cr alloy had a lower i_corr_ and a higher R_p_ at pH 6.8 (close to neutral), which means it performed better at surviving corrosion.
- At pH 4.9 (acidic), the SLM alloy showed a higher electropositive E_oc_ (and E_corr_ trend), which means it worked better in acidic conditions. However, this benefit may be cancelled out by process-related porosity/particulates unless surface optimization is used.
- Increasing the pH reduced i_corr_ values for both alloys, which is in line with better passivation.
- In a clinical setting, choosing the right materials and optimizing the surface for SLM should take into account the acidic exposures that are unique to each patient (diet, reflux, biofilm).

## Data Availability

Further data is available from the corresponding author upon reasonable request.
